# Serum Levels of HMGB1, hS100A8/A9, and sRAGE in Patients with Knee and Hip Osteoarthritis: Inflammatory Biomarkers of Disease Activity

**DOI:** 10.3390/jcm14175931

**Published:** 2025-08-22

**Authors:** Sandra Rusac-Kukić, Alenka Višnić, Maja Rogić Vidaković, Dubravka Bobek

**Affiliations:** 1 Department of Physical Medicine and Rehabilitation, Special Hospital for Medical Rehabilitation of Heart, Lung and Rheumatic Diseases, Thalassotherapia Opatija, 51410 Opatija, Croatia; sandra.rusac-kukic@tto.hr; 2 Department of Human Reproduction, Clinic for Gynecology and Obstetrics, Clinical Hospital Center Rijeka, 51000 Rijeka, Croatia; 3 Laboratory for Human and Experimental Neurophysiology (LAHEN), Department of Neuroscience, School of Medicine, University of Split, 21000 Split, Croatia; maja.rogic@mefst.hr; 4 Department of Physical Medicine and Rehabilitation, University Hospital Dubrava, 10000 Zagreb, Croatia; procelnik.fizik@kbd.hr

**Keywords:** biomarkers, hip, HMGB1, inflammation, knee, osteoarthritis, sRAGE, hS100A8/A9

## Abstract

**Background/Objectives:** Osteoarthritis (OA) is the most prevalent type of arthritis, primarily impacting synovial joints. While it has traditionally been viewed as resulting from mechanical wear and tear, OA is now increasingly understood as an inflammatory condition. By analysing serum concentrations of molecular patterns related to inflammatory damage (DAMPs), including high-mobility group box 1 protein (HMGB1), hS100A8/A9 proteins, and their soluble receptor for advanced glycation end products (sRAGE), it is possible to investigate the inflammatory pathogenesis of the disease. **Methods:** The research was conducted at Dubrava University Hospital in Zagreb, Croatia, from March 2022 to July 2024. The study analyses venous blood from 94 subjects with different degrees of knee osteoarthritis (KOA) and hip osteoarthritis (hip OA) using ELISA. The aim is to measure serum levels of DAMP biomarkers, including HMGB1, hS100A8/A9, and sRAGE. **Results:** Subjects with KOA exhibited higher levels of HMGB1 (21.72 ± 3.50) than those with hip OA (7.11 ± 1.46) or controls (1.64 ± 0.80), indicating a potential role for HMGB1 as a proinflammatory mediator. Lower sRAGE levels in KOA (499.97 ± 18.71) compared to controls (1273.8 ± 58.92) may suggest impaired anti-inflammatory activity. Because of possible differences in biomechanical loads and metabolic pathways, hS100A8/A9 concentrations in KOA (1227.06 ± 175.34) were greater than in hip OA (664.88 ± 38.90). **Conclusions:** HMGB1 exhibits proinflammatory and sRAGE anti-inflammatory activity in knee osteoarthritis (KOA) and hip OA. Their levels support an inflammatory pathogenesis of these diseases. HMGB1 and sRAGE are promising biomarkers for monitoring disease progression and could represent potential therapeutic targets.

## 1. Introduction

Osteoarthritis (OA) is the most prevalent degenerative joint disease, primarily affecting individuals over the age of 65, and it occurs more frequently in women than in men. Clinically, OA is believed to result from a combination of various risk factors: genetics, age, gender, obesity, joint trauma, altered biomechanics, and coexisting metabolic diseases, along with other comorbidities [1]. The effects of OA include pain and reduced mobility in the affected joint, which can lead to disability and a lower quality of life [2]. OA is a musculoskeletal disorder characterised by progressive cartilage deterioration, synovial inflammation, and alterations in subchondral bone [3]. It most commonly affects the knee joint structures, including the subchondral bone, cartilage, synovial membrane, meniscus, ligaments, and adipose tissue. The infrapatellar (IFP) and suprapatellar (SFP) fat pads are structures inside the knee that assist with joint movement and also contribute significantly to the development and progression of knee osteoarthritis (KOA) [1].

Historically, OA has been regarded as a non-inflammatory form of arthritis; however, the distinction between inflammatory and degenerative arthritis is becoming less clear, as mounting evidence indicates increased immune activity in the joints and synovium caused by OA. Synovitis is the inflammation of the synovial membrane, commonly associated with inflammatory arthritis. Additionally, various soluble mediators, including cytokines and prostaglandins, can induce matrix metalloproteinase (MMP) production in chondrocytes. MMP is closely linked to the diagnosis of primary OA and is regarded as a contributing factor to the disease’s progression. This process expedites the initial inflammatory phase associated with synovitis, a critical aspect of OA that markedly influences its progression [4]. Synovitis occurs in a considerable number of OA patients and is linked to the pathological processes involved in the progression of OA [5].

The innate immune system recognises endogenous molecules released by damaged or dying cells. These are known as damage-associated molecular patterns (DAMPs), which can signal sterile cell injury caused by chemical toxins, burns, trauma, or hypoxia [6]. The concept of inflammatory pathogenesis of OA is increasingly recognised as a crucial factor, with DAMPs playing a central role in initiating and sustaining low-grade inflammatory responses [7,8]. Although there is no treatment to halt or prevent OA, advances have been made in understanding its pathophysiological mechanisms. Current knowledge about OA as an inflammatory disease highlights the key role of innate immunity in this process. Pattern recognition receptors (PRRs), which detect DAMPs and initiate inflammation, are essential components of the innate immune system [9,10]. Proteins released from stressed, injured, or dead cells act as a critical source of DAMPs [11]. DAMPs are usually located inside cells and can initiate immune responses when released, such as activating high-mobility group box 1 (HMGB1) [12]. HMGB proteins have multifunctional roles, acting as innate and endogenous regulators of joint inflammation. Additionally, they work together in cartilage hypertrophy and support the maintenance of joint tissue homeostasis [13,14].

HMGB1 is a high-mobility group protein (HMG) that interacts with DNA inside the nucleus. Once released outside the cell, it attracts osteoclasts, osteoblasts, and endothelial cells, thereby boosting the inflammatory response. HMGB1 can be released either actively or passively; inflammatory triggers promote its movement from the nucleus to the cytoplasm and then outside the cell, while necrosis and apoptosis lead to its passive release [15]. This essential inflammatory signalling molecule activates multiple intracellular pathways, leading to the production of cytokines, chemokines, and metalloproteinases. HMGB1 facilitates the disease process by stimulating synovial fibroblasts, macrophages, and chondrocytes through receptors such as RAGE and TLR. This stimulation promotes the release of proinflammatory cytokines like IL-1β, TNF-α, and MMPs, which contribute to the breakdown of articular cartilage [16]. The molecular mechanism of pyroptosis in fibroblast-like synoviocytes (FLSs) and the release of HMGB1 involves the mediated activation of cytokine release (IL-1β, TNF-α) by HMGB1 from synovial fibroblasts and macrophages [17,18].

Toll-like receptors (TLRs) and receptors for advanced glycation end products (RAGEs) are also vital in the inflammation and catabolic processes that damage cartilage [19,20]. Specific proteins, such as S100A4, A8, A9, and A11, have been recognised as inflammatory markers because of their ability to activate sRAGE and TLRs [21]. Advanced glycation end products (AGEs) form through the non-enzymatic glycation of proteins, lipids, and amino acids. When AGEs bind to the receptor for advanced glycation end products (RAGE), they can initiate chronic inflammation and oxidative stress by activating multiple cellular signalling pathways. This results in increased reactive oxygen species (ROS) levels and the recruitment of proinflammatory cells [22,23]. RAGE plays a crucial role in the innate immune response and functions as a mediator of proinflammatory processes, binding to molecules from stressed or damaged cells [24].

hS100A8/A9, also known as calprotectin, plays a vital role in OA by promoting synovial inflammation and cartilage damage. It is released by activated synovial macrophages, neutrophils, and chondrocytes within affected joints, acting as a damage-associated molecular pattern (DAMP) [25]. The alarmins hS100A8 and hS100A9 have long been recognised as markers of joint destruction. Previously referred to as MRP8 and MRP9 (myeloid-related proteins), these molecules are present at high levels in synovial fluid, signalling leukocyte activity and contributing to the progression of joint damage. Recent studies demonstrate that these alarmins actively mediate inflammation, signalling through the TLR4 receptor. By engaging TLR4 and RAGE receptors, they activate the NF-κB and MAPK (mitogen-activated protein kinase) pathways. This activation stimulates chondrocytes to increase production of matrix metalloproteinases, which speeds up cartilage breakdown and decreases the synthesis of type II collagen and aggrecan, thereby hindering the repair and regeneration of damaged cartilage [26]. Additionally, it enhances osteoclast activity, resulting in increased bone resorption and sclerosis in the later stages of the disease [27]. Furthermore, hS100A8/A9 activates nociceptive neurons through RAGE, thus contributing to chronic OA pain [6].

A study in mice has shown that monocytes are the initial cells in the inflamed synovium of OA. Moreover, local induction of OA leads to a notable increase in hS100A8/A9 expression and promotes the recruitment of monocytes from the bone marrow to the synovium [28]. Research increasingly emphasises the role of S100 family proteins in the inflammation associated with OA [29,30,31]. Defensins are another group of DAMPs associated with catabolic responses in OA, with increased levels of β-defensin observed in cartilage and menisci affected by OA [32]. Additionally, monosodium crystals—key inflammatory triggers in gout—may worsen OA by triggering inflammation and releasing interleukins and other DAMPs [33]. Experimental data suggest that subchondral bone could be key in the OA process, acting as a mechanical buffer and a source of inflammatory mediators that contribute to OA and deep cartilage breakdown [4,34].

Immunological parameters have a more crucial role in diagnosing OA compared to biochemical and physiological parameters [35]. The age-related accumulation of DAMPs highlights the importance of exploring PRRs, particularly TLRs and RAGE, as potential targets for therapy of OA. Crucial RAGE ligands, like HMGB1 and S100 proteins, gather with age, contributing to ongoing low-level, sterile inflammation [9,11]. OA was traditionally regarded as an age-related condition characterised by cartilage degradation. However, OA involves more than changes linked to age; it is a complex disorder affecting all joint components and is associated with a series of proinflammatory responses that drive disease progression [36].

This study aims to clarify the inflammatory mechanisms behind KOA and hip OA by examining serum levels of damage-associated molecular patterns (DAMPs), with special attention given to HMGB1, hS100A8/A9 proteins, and sRAGE.

## 2. Materials and Methods

### 2.1. Ethics

Approval for this study was granted by the Ethics Committee of the tertiary Faculty of Medicine, University of Rijeka, Croatia (ethical approval No.: 003-08/21-01/20). All subjects provided informed consent.

### 2.2. Patient Characteristics

The research was carried out at the Departments of Physical and Rehabilitation Medicine, Traumatology, and Orthopaedics of Dubrava University Hospital in Zagreb, Croatia, from March 2022 to July 2024.

Subjects in the study group (*n* = 94) were diagnosed with mild to severe OA affecting either the hip or knee. Diagnosis was based on medical history, clinical examination, laboratory results, and X-ray evaluation of KOA and hip OA. The stage of KOA and hip OA progression was assessed using radiography and the Kellgren–Lawrence grading scale. Pain levels in all patients were measured using the visual analogue scale (VAS), the Western Ontario and McMaster University Arthritis Index (WOMAC), and according to their self-reported symptoms. Participants were divided into four subgroups based on the confirmed OA stages: subgroup one—hip OA stages I and II (*n* = 25); subgroup two—KOA stages I and II (*n* = 23); subgroup three—hip OA stages III and IV (*n* = 24); subgroup four—KOA stages III and IV (*n* = 22).

The control group (*n* = 96) consisted of subjects who did not have OA or any other diseases or conditions that could affect the analytical outcomes. Participants in the control group did not show elevated laboratory values for erythrocyte sedimentation rate (ESR) or C-reactive protein (CRP) at the time of blood sampling. They also exhibited no signs or symptoms of hip OA, KOA, or hand OA, nor did they have recent musculoskeletal injuries or inflammatory rheumatic diseases.

Exclusion criteria include neurodegenerative and neuroinflammatory diseases, diabetes mellitus and its complications, autoimmune inflammatory diseases, inflammatory rheumatic disease, degenerative bone disorders, concurrent symptoms of hip OA and KOA, elevated inflammatory laboratory values (SE, CRP), hand OA, chronic kidney diseases, post-traumatic OA, and other metabolic diseases.

### 2.3. Samples

Peripheral venous blood samples were analysed from all study participants and the control group between March 2022 and July 2024. The clinical portion of the study was conducted at the Department of Physical and Rehabilitation Medicine with Rheumatology and the Department of Orthopaedics and Traumatology at the University Hospital Dubrava, Zagreb, Croatia. Laboratory analyses were carried out at the Clinical Department of Laboratory Diagnostics of the University Hospital Centre Zagreb, Zagreb, Croatia.

For serum biochemical analysis, 6 mL of blood was collected by venipuncture between 9 and 10 a.m. For analyses using turbidimetry and EIA, blood was collected in tubes containing sodium EDTA as an anticoagulant, while for the ELISA method, blood was collected in tubes without EDTA (BD Vacutainer Systems—7 mL, Plymouth, UK; Cat. No. 367896). Blood samples were centrifuged at room temperature for 5 min at 3500 rpm (Hettich Centrifuge, Universal 320/320 R, Andreas Hettich GmbH & Co., Tuttlingen, Germany; operated with HettInfo II Software version 1.2, Hettich, Germany). The supernatant (plasma) was separated and stored in cryotubes (Nunc CryoTubes, Thermo Fisher Scientific, Roskilde, Denmark) at −70 °C until analysis. In purified blood samples, the protein concentration was determined using the Bradford method, as described by Kielkopf [37]. The CRP concentration was analysed using the EIA technique, according to Salonen [38]. The ELISA method was performed following the manufacturer’s instructions to assess plasma protein levels of HMGB1, hS100A8/9, and their sRAGEs: RAGE (R&D Systems, Minneapolis, MN, USA); S100A12/EN-RAGE (CycLex Co., Nagano, Japan); and HMGB1 (Shino-Test Corporation, Tokyo, Japan). The sensitivities of the ELISA were 1 ng/mL for HMGB1, 56 pg/mL for S100, and 4.12 pg/mL for RAGE (SoftMax Pro Software version 7.0.3, SpectraMax iD3, Molecular Devices, San Jose, CA, USA). Serum concentrations of the laboratory parameters were measured, including erythrocyte sedimentation rate, haematocrit, haemoglobin, peripheral blood platelets, and leukocytes, using turbidimetry (Abbott Alinity c analyzer, Abbott Laboratories, Abbott Park, IL, USA, operated with AlinIQ Software version 3.0, Abbott Diagnostics, Chicago, IL, USA), as described in the study by Correia [39].

### 2.4. Statistical Analysis

The descriptive analysis of the relevant data was summarised using the number of observations (*n*), percentages, means (Ms), and standard deviations (SDs). To identify differences in specific variables, Student’s *t*-test, chi-square test (χ^2^), and Mann–Whitney U test (Z value) were utilised. A significance level of *p* < 0.05 was considered statistically significant. Data analysis was conducted using StatSoft version 12.

## 3. Results

The study included 94 participants in the experimental group and 96 in the control group, with an equal number of men and women aged between 51 and 75 years. The average age was 64 for women and 66 for men. All participants hailed from Croatia’s continental region and had a middle socioeconomic status. None were pathologically obese, with a BMI exceeding 40 kg/m^2^.

**Figure 1:**
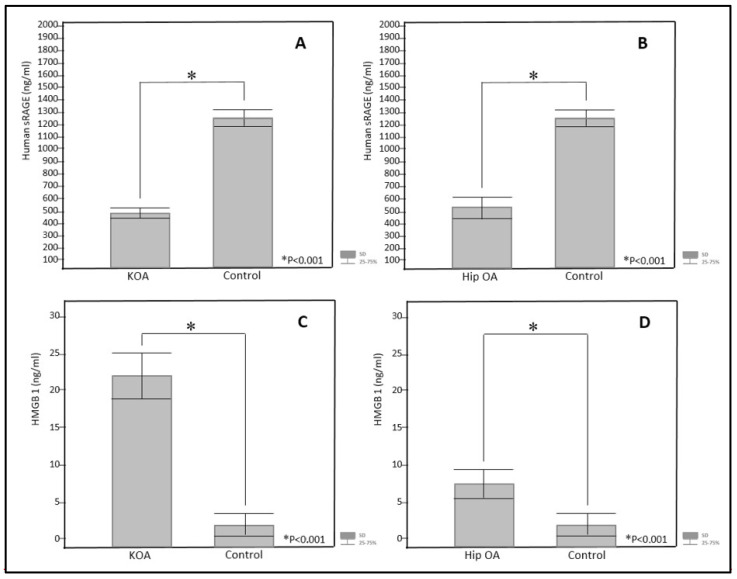
Serum concentrations of human sRAGE in subjects with KOA (**A**) and hip OA (**B**), and HMGB1 in subjects with KOA (**C**) and hip OA (**D**), compared to the control group. HMGB1 = high-mobility group box one protein; sRAGE = soluble receptor for advanced glycation end products; hip OA = hip osteoarthritis; KOA = knee osteoarthritis. Statistical methods: chi-square test (χ^2^) and Mann–Whitney U test (Z value); StatSoft 12 software. * Statistical significance *p* < 0.001.

The distribution of participants by gender (χ^2^ = 0.27) and age (t = −1.56) did not show statistically significant differences between subjects with KOA and hip OA compared to the control group (Table 1). Globulin analysis revealed an important difference, with subjects with KOA having higher levels of alpha 1 globulin, alpha 2 globulin, beta globulin, and gamma globulin compared to subjects with hip OA (Table 2). Additionally, HMGB1 and hS100A8/A9 protein concentrations were significantly higher in subjects with KOA compared with those with hip OA, but showed no significant difference compared with controls. HMGB1 levels increased with disease progression in both groups, while hS100A8/A9 levels did not display such an increase (Table 3 and Table 4, and Figure 1). Conversely, sRAGE concentrations did not differ between subjects with hip OA and KOA; however, they were significantly lower in the study group compared to the control group, and they did not change with the progression of disease stage in either group (Table 3 and Table 4, and Figure 1).

## 4. Discussion

In this study, HMGB1 and human hS100A8/A9 levels showed significant differences between subjects with KOA and hip OA. Furthermore, HMGB1 is linked to disease progression, highlighting its potential as a non-invasive biomarker for diagnosis and treatment monitoring. In contrast, the levels of hS100A8/A9 and sRAGE proteins remain steady across all OA stages (grades 1–4). The levels of HMGB1, and sRAGE are significantly different between the OA group and the control group. These findings highlight the importance of damage-associated molecular patterns (DAMPs) in OA’s pathophysiology, indicating that OA is associated with low-grade inflammation and deepening our understanding of the disease. Many studies have shown an association between HMGB1 and sRAGE in OA; however, the link between their concentrations and disease progression remains under investigation, emphasising the importance of this study.

### 4.1. High-Mobility Group Box 1 (HMGB1)

Elevated levels of HMGB1 are associated with the inflammatory progression of OA, supporting the concept that this proinflammatory alarmin functions as a mediator in the innate immune response and might be involved in the disease’s immunopathogenesis [16].

Existing evidence indicates that HMGB1 is released from damaged or dying cells in the joint, including chondrocytes and synovial cells [15]. Additionally, elevated levels of HMGB1 promote cartilage matrix breakdown by inducing enzymes such as MMP-130. Extracellular HMGB1 plays a significant role in the development of synovitis, a common condition in KOA and hip OA, exacerbating joint degeneration. This process highlights the essential role of the immune system, particularly HMGB1, in the immunopathogenic mechanisms associated with OA. The hip joint bears greater weight but has a more stable structure with higher load capacity. These features of hip OA, combined with reduced microtrauma and more stable biomechanics, lead to less cellular damage [7,40,41]. However, OA is more pronounced in KOA, with elevated HMGB1 levels [17,18]. Analysis of HMGB1 concentrations in our study also showed significantly higher levels in KOA compared to hip OA. These findings confirm that more unstable biomechanics in the knee joint contribute to greater damage to the joint structure and the development of KOA. Jiang [42] states that every 5-unit rise in BMI results in a 35% increased risk of KOA, highlighting a positive relationship between higher BMI and the likelihood of developing this condition. Although our study did not track the BMI of participants, none had a BMI over 40 kg/m^2^. However, obese individuals showed a higher likelihood of having KOA, supporting previous research.

HMGB-1 levels are higher in the synovial membrane of individuals with KOA. Elevated HMGB-1 in the synovial fluid relates to more severe synovitis, pain, and daily functional issues. This suggests that HMGB-1, a proinflammatory cytokine, may have a crucial role in KOA progression [43]. The diagnosis of hip OA and KOA in our study was determined through clinical and laboratory findings, combined with X-ray assessment of KOA and hip OA. According to these criteria, respondents were divided into four groups (grades 1–4). Although subjects did not report more severe pain in KOA than in hip OA as the disease advanced, i.e., in higher stages (grades 3–4), their pain was significantly more intense than in the earlier stages (grades 1–2), along with elevated HMGB1 concentrations. Our research confirms these findings while also indicating that HMGB1 levels increase as the disease advances, implying a connection to the degree of joint tissue damage. Although earlier studies have established HMGB1’s role in OA [44,45,46,47], our research highlights its potential use in assessing disease severity, particularly in KOA.

### 4.2. Human sRAGE

RAGE is found in the cell membrane, and the soluble form of this receptor that lacks the cytoplasmic and transmembrane domains is called soluble RAGE (sRAGE). It acts as a decoy receptor, reducing inflammation by binding RAGE ligands and preventing their interaction with membrane-bound RAGE. sRAGE may also help limit oxidative stress, a crucial factor in the development of OA. Variations in sRAGE levels can be affected by genetic differences between individuals, and systemic factors such as diet also play a significant role, which may lead to decreased activation of inflammatory pathways in certain joints and have different effects on the knee and hip joints [24].

Chayanupatkul and Honsawek [48] conducted a study to assess the severity of OA using the Kellgren–Lawrence grading system. Their analysis of sRAGE levels in plasma and synovial fluid from OA patients showed that plasma sRAGE levels were significantly lower than those in healthy controls. A positive correlation was observed between sRAGE levels in plasma and synovial fluid. Multiple studies analysing plasma and synovial fluid in OA have demonstrated that sRAGE levels are reduced compared to healthy controls [44,47]. Levels of plasma and synovial fluid sRAGE decrease as disease severity increases [48,49]. Our study found that subjects with hip OA and KOA had lower serum sRAGE concentrations compared to control subjects, which aligns with previous research. The difference in serum levels between those with hip and knee involvement was not statistically significant. Although sRAGE is known to be protective in inflammatory conditions, and in this study, HMGB1 was significantly higher in KOA than in hip OA, sRAGE levels did not correlate with HMGB1 concentrations or the severity of inflammation. There was also no significant difference in sRAGE concentrations across different stages of OA (grades 1–4), confirming the variability in its levels and the degree of disease. All this supports the previous study [24] that suggested many factors influence sRAGE concentrations.

### 4.3. Human hS100A8/9

hS100A8/9 is a key pain mediator released from inflamed synovium during the acute inflammatory phase in the joint [50]. In a study by Mahler [51], serum hS100A8/A9 levels showed no significant differences between patients with KOA and hip OA, and no link was found between these levels and clinical features. However, serum hS100A8/A9 was negatively associated with total osteophyte count and disease progression. The inverse relationship with structural abnormalities and the positive correlation with erythrocyte sedimentation rate (ESR) may suggest inflammatory processes in the synovium before structural changes. Multiple studies have examined hS100A8/A9 in KOA and hip OA, finding elevated levels of these proteins [16,28,52].

In our study, we partially confirmed the results of previous research. We observed elevated levels of hS100A8/A9 in patients with OA of the KOA, but not in subjects with hip OA compared to controls. However, despite these findings, it was noted that hS100A8/A9 levels were twice as high in subjects with KOA compared to those with hip OA, which has not been reported before. hS100A8/A9 proteins are known to mediate inflammation actively and can accelerate cartilage degradation, having a crucial role in its destruction [26]. Although it would be expected that their concentrations increase with disease progression, we did not find significant differences in serum levels across the different stages of OA (stages 1–4).

Validation of biomarkers in clinical samples is a complex challenge that involves biological, technical, and clinical factors. Significant issues include patient heterogeneity (age, gender, comorbidities), variations in disease stage and progression, and preanalytical variability related to sample processing, storage, and type [53].

Additional challenges involve the absence of reference values, variability across analytical methods, and a limited connection between biomarkers and clinically meaningful outcomes [54,55,56,57].

Understanding the clinical significance of biomarkers such as HMGB1, hS100A8/A9, and sRAGE could facilitate personalised treatment strategies for OA. Elevated HMGB1 levels and decreased sRAGE levels may serve as signals of active disease, allowing the identification of patients with an inflammatory phenotype of OA who could benefit from early anti-inflammatory or disease-modifying therapies, especially with the emergence of new biologics that target alarmin and DAMP-mediated signalling pathways [15,17,58].

Study limitations: A larger study is needed to assess changes in biomarker levels over time, as current data are restricted to a single time point. Moreover, the study was confined to one geographic area, and factors such as physical activity and body mass index (BMI) were not investigated. The study’s strengths include the use of matched controls, validated analytical methods, and the concurrent analysis of three complementary biomarkers: HMGB1, sRAGE, and hS100A8/A9 proteins.

## 5. Conclusions

Elevated serum levels of HMGB1 and reduced levels of hS100A8/A9 proteins in individuals with KOA and hip OA suggest their potential role in the disease’s immunopathology. The link between HMGB1, disease severity, and clinical outcomes suggests that HMGB1 could be beneficial in diagnosis, tracking disease progression, and evaluating treatment response. As biological testing and personalised medicine advance, this biomarker may become crucial in future OA management.

## Figures and Tables

**Table 1:** Demographic and clinical characteristics of subjects with hip OA and KOA.

	Hip OA (M ± SD)	KOA (M ± SD)	*p*-Value
*n*	49	45	-
Female/Male (*n*)	27/22	26/19	0.60
Age (range)	51–75	51–74	-
Age (years)	63.00 ± 6.04	64.91 ± 5.74	0.73
VAS pain	4.93 ± 1.79	5.50 ± 2.37	0.07
WOMAC pain	9.84 ± 3.18	9.02 ± 4.68	0.41
WOMAC stiffness	4.08 ± 1.63	3.28 ± 2.00	0.10
WOMAC function	33.06 ± 11.95	31.40 ± 13.80	0.68

Hip OA = hip osteoarthritis; KOA = knee osteoarthritis; VAS = visual analogue pain scale; WOMAC = Western Ontario and McMaster University Arthritis Index. Statistical method: Student’s *t*-test, chi-square test (χ^2^), and Mann–Whitney U test (Z value); StatSoft 12 software.

**Table 2:** Laboratory findings in patients with hip OA and KOA.

	Hip OA (M ± SD)	KOA (M ± SD)	*p*-Value
ESR (mm/h)	17.87 ± 9.84	19.25 ± 15.20	0.84
CRP (mg/L)	2.73 ± 1.93	7.10 ± 19.75	0.70
Haematocrit (L/L)	0.41 ± 0.04	0.42 ± 0.04	0.79
Platelets × 10^9^/L	245.52 ± 61.61	260.95 ± 58.58	0.14
Leukocytes × 10^9^/L	7.06 ± 2.48	7.21 ± 2.44	0.81
Albumin (g/L)	43.38 ± 4.31	48.89 ± 9.30	0.72
Alpha 1 globulin (g/L)	2.92 ± 0.43	5.45 ± 0.74	<0.001 *
Alpha 2 globulin (g/L)	4.35 ± 1.16	9.15 ± 1.88	<0.001 *
Beta globulin (g/L)	8.11 ± 1.05	11.66 ± 1.79	<0.001 *
Gamma globulin (g/L)	7.56 ± 1.91	13.41 ± 5.51	<0.001 *
A/G ratio	1.51 ± 0.21	1.42 ± 0.24	0.10

Hip OA = hip osteoarthritis; KOA = knee osteoarthritis; ESR = erythrocyte sedimentation rate; CRP = C-reactive protein; A/G ratio = albumin-to-globulin ratio. Statistical method: Student’s *t*-test, chi-square test (χ^2^), and Mann–Whitney U test (Z value); StatSoft 12 software. * Statistical significance *p* < 0.05.

**Table 3:** Concentrations of sRAGE, HMGB1, and hS100A8/A9 in patients with hip OA and KOA, and control subjects.

	Hip OA (M ± SD)	KOA (M ± SD)	*p*-Value
sRAGE (ng/mL)	566.76 ± 22.89	499.97 ± 18.71	0.84
HMGB1 (ng/mL)	7.11 ± 1.46	21.72 ± 3.50	<0.001 *
hS100A8/A9 (ng/mL)	664.88 ± 38.90	1227.06 ± 175.34	<0.001 *
	**Hip OA (M ± SD)**	**Control (M ± SD)**	***p*** **-Value**
sRAGE (ng/mL)	566.76 ± 22.89	1273.87 ± 58.92	<0.001 *
HMGB1 (ng/mL)	7.11 ± 1.46	1.64 ± 0.80	<0.001 *
hS100A8/A9 (ng/mL)	664.88 ± 38.90	916.21 ± 123.13	0.24
	**KOA (M ± SD)**	**Control (M ± SD)**	***p*** **-Value**
sRAGE (ng/mL)	499.97 ± 18.71	1273.8 ± 58.92	<0.001 *
HMGB1 (ng/mL)	21.72 ± 3.50	1.64 ± 0.80	<0.001 *
hS100A8/A9 (ng/mL)	1227.06 ± 175.34	916.21 ± 123.13	0.55

Hip OA = hip osteoarthritis; KOA = knee osteoarthritis; sRAGE = soluble receptor for advanced glycation end products; HMGB1 = high-mobility group box one protein; hS100A8/A9 = human proteins S100A8/A9. Statistical method: chi-square test (χ^2^), and Mann–Whitney U test (Z value); StatSoft 12 software. * Statistical significance *p* < 0.05.

**Table 4:** HMGB1 concentrations depending on disease stage in hip OA and KOA.

	Grade 1 *n* = 9 (M ± SD)	Grade 2 *n* = 13 (M ± SD)	Grade 3 *n* = 12 (M ± SD)	Grade 4 *n* = 11 (M ± SD)	
	**HMGB1** **ng/mL**	**HMGB1** **ng/mL**	**HMGB1** **ng/mL**	**HMGB1** **ng/mL**	***p*-Value**
KOA	8.93 ± 1.32	18.64 ± 2.04	21.84 ± 2.68	35.68 ± 3.47	<0.01 *
Hip OA	5.51 ± 0.23	8.24 ± 1.06	11.46 ± 1.37	16.57 ± 2.71	<0.01 *
	**hS100A8/A9** **ng/mL**	**hS100A8/A9** **ng/mL**	**hS100A8/A9** **ng/mL**	**hS100A8/A9** **ng/mL**	***p*-Value**
KOA	971.03 ± 124.71	832.8 ± 97.23	901.84 ± 98.69	1106.21 ± 134.77	0.86
Hip OA	498.81 ± 31.25	576.24 ± 87.42	621.21 ± 67.14	613.49 ± 97.87	0.72
	**sRAGE** **ng/mL**	**sRAGE** **ng/mL**	**sRAGE** **ng/mL**	**sRAGE** **ng/mL**	***p*-Value**
KOA	369.69 ± 29.66	501.98 ± 39.41	498.84 ± 46.74	572.34 ± 21.39	0.81
Hip OA	481.81 ± 73.81	612.15 ± 46.28	591.76 ± 23.57	673.97 ± 31.15	0.68

HMGB1 = high-mobility group box one protein; hS100A8/A9 = human proteins S100A8/A9; sRAGE = soluble receptor for advanced glycation end products; hip OA = hip osteoarthritis; KOA = knee osteoarthritis. KOA and hip OA are classified into 4 stages (1 = mildest; 4 = most severe). Statistical methods: chi-square test (χ^2^) and Mann–Whitney U test (Z value); StatSoft 12 software. * Statistical significance *p* < 0.05.

## Data Availability

All data generated and analysed in the presented study are included in this published article.
